# Ozone profiles in the Baltimore-Washington region (2006–2011): satellite comparisons and DISCOVER-AQ observations

**DOI:** 10.1007/s10874-014-9283-z

**Published:** 2014-05-14

**Authors:** Anne M. Thompson, Ryan M. Stauffer, Sonya K. Miller, Douglas K. Martins, Everette Joseph, Andrew J. Weinheimer, Glenn S. Diskin

**Affiliations:** 1 Department of Meteorology, Pennsylvania State University, 503 Walker Building, University Park, PA 16802-5013 USA; 2 Present Address: NASA/Goddard Space Flight Center, Code 614, Greenbelt, MD 20771 USA; 3 Department of Physics and Astronomy, Howard University, 2355 Sixth Street NW, Washington, DC 20059 USA; 4 Atmospheric Chemistry Division, NCAR, Boulder, CO 80307 USA; 5 NASA Langley Research Center, MS 401B, Hampton, VA 23681 USA

**Keywords:** Tropospheric ozone, Stratosphere-troposphere exchange, Satellite validation, DISCOVER-AQ, Baltimore pollution, Ozonesondes, Washington DC pollution, Air quality, Carbon monoxide, Aircraft chemical measurements

## Abstract

Much progress has been made in creating satellite products for tracking the pollutants ozone and NO_2_ in the troposphere. Yet, in mid-latitude regions where meteorological interactions with pollutants are complex, accuracy can be difficult to achieve, largely due to persistent layering of some constituents. We characterize the layering of ozone soundings and related species measured from aircraft over two ground sites in suburban Washington, DC (Beltsville, MD, 39.05 N; 76.9 W) and Baltimore (Edgewood, MD, 39.4 N; 76.3 W) during the July 2011 DISCOVER-AQ (Deriving Information on Surface Conditions from Column and Vertically Resolved Observations Relevant to Air Quality) experiment. First, we compare column-ozone amounts from the Beltsville and Edgewood sondes with data from overpassing satellites. Second, processes influencing ozone profile structure are analyzed using Laminar Identification and tracers: sonde water vapor, aircraft CO and NO_y_. Third, Beltsville ozone profiles and meteorological influences in July 2011 are compared to those from the summers of 2006–2010. Sonde-satellite offsets in total ozone during July 2011 at Edgewood and Beltsville, compared to the Ozone Monitoring Instrument (OMI), were 3 % mean absolute error, not statistically significant. The disagreement between an OMI/Microwave Limb Sounder-based tropospheric ozone column and the sonde averaged 10 % at both sites, with the sonde usually greater than the satellite. Laminar Identification (LID), that distinguishes ozone segments influenced by convective and advective transport, reveals that on days when both stations launched ozonesondes, vertical mixing was stronger at Edgewood. Approximately half the lower free troposphere sonde profiles have very dry laminae, with coincident aircraft spirals displaying low CO (80–110 ppbv), suggesting stratospheric influence. Ozone budgets at Beltsville in July 2011, determined with LID, as well as standard meteorological indicators, resemble those of 4 of the previous 5 summers. The penetration of stratospheric air throughout the troposphere appears to be typical for summer conditions in the Baltimore-Washington region.

## Introduction

Although ground-level ozone is a routinely monitored pollutant in the US, important information about regional and intra- and inter-regional transport comes from measurements of ozone profiles because they encompass the levels in the middle and lower troposphere at which transport actually occurs. A multi-decadal record of ozone profiles taken by ozonesondes in the US exists for two sites only (Boulder, Colorado, 40 N, 105 W; Wallops Island, Virginia, 37.85 N, 75.5 W). These records have been augmented since the 1990’s at Huntsville, Alabama, typically not in the reach of an urban environment, and Trinidad Head, California, a station downwind of growing Asian emissions (Cooper et al. 2011). Since 2004 two urban regions have been sampled with some regularity during their ozone seasons: Houston, Texas, (Morris et al. 2006; Morris et al. 2010; Rappenglueck et al. 2008) and Washington, DC (Thompson et al. 2007a; Thompson et al. 2007b; Morris et al. 2007; Yorks et al. 2009). Influences on ozone include local pollution, long-range transport with occasional reaches from Canadian and Alaskan fires, and frequent enrichment by air of stratospheric origins. In the case of Washington, DC, these conclusions are consistent with summertime aircraft profiles over Maryland (Taubman et al. 2006; Hains et al. 2008).

A larger mid-Atlantic perspective on tropospheric ozone comes from Wallops Island (Newchurch et al. 2003; Normile et al. 2014, in review) and from the 2009 and 2010 CAPABLE (Chemistry and Physics of the Atmospheric Boundary Layer Experiment) soundings in Hampton, Virginia (37.1 N, 76.3 W). The latter observations are complemented by continuous surface ozone readings; the bay breeze effect from the adjacent Chesapeake Bay was isolated as an important component of ozone exceedance in the Hampton data (Martins et al. 2012).

The 2011 DISCOVER-AQ (Deriving Information on Surface Conditions from Column and Vertically Resolved Observations Relevant to Air Quality) campaign put mid-Atlantic air quality (AQ) under a microscope, so to speak, during a polluted July, with ozone profiles measured by aircraft (NASA P-3, University of Maryland Cessna, NASA UC-12 with remote sensing instruments), frequent soundings in suburban Washington and Baltimore, and deployment of additional instrumentation at six EPA-certified monitoring sites maintained by the Maryland Department of Environment (MDE). The instruments, for which data at Edgewood and Beltsville, Maryland, are used in the present analysis, are listed in Table 1. They served a range of goals, from satellite validation, intercomparison of selected observations, flux determination, elucidation of boundary-layer structure over a diurnal cycle and the characterization of volatile organic compounds (VOC)-NO_x_ relationships in an area with complex natural and anthropogenic sources of ozone precursors.

**Table 1:** Instruments and data sets used

Instrument/product	Data	Origin of data
OMI	Total Column O_3_, NO_2_	Aura Satellite
		http://avdc.gsfc.nasa.gov/index.php?site=666843934&id=13
TTOR	Tropospheric Column O_3_ with GSFC trajectories enhancing MLS & OMI	ftp://hyperion.gsfc.nasa.gov/pub/aura/tropo3
ECC Ozonesondes, Radiosondes	Total, tropospheric Column O_3_	Edgewood, MD
		Beltsville, MD
Ozone analyzer	Continuous surface O_3_	Edgewood, MD (NATIVE Thermo 49c)
		Beltsville, MD (MDE Thermo 49i)
NCAR Ozone-NO/NOy Analyzer	NO, NO_y_	P-3 Spirals over Beltsville and Edgewood, MD
	Fast Response O_3_	A J Weinheimer, PI
NASA/LaRC CO DACOM	CO	P-3 Spirals over Beltsville and Edgewood, MD
		G S Diskin, PI; Sachse et al. 1987

In this study we focus on ozone soundings taken during DISCOVER-AQ at the Beltsville, Maryland (39.05.N, 76.9 W) location, 20 km NE of downtown Washington, DC, and at Edgewood, Maryland (39.4 N; 76.3 W), 35 km NE of downtown Baltimore. Surface ozone, NO_y_ (reactive nitrogen consisting of NO + NO_2_ + HNO_3_ + PAN + organic nitrates), NO and CO measurements were made continuously at both sites; selected VOC data are also available (Halliday et al. 2013). During P-3 flight days, fast ozone measurements were collected in aircraft spirals above Beltsville and Edgewood 3–4 times, with one or two of the spirals coinciding with the soundings. Detailed comparisons of NO/NO_2_/NO_y_ (Reed et al. 2013; Stauffer et al. 2012) and ozone (Martins et al. 2013) among ground-based, balloon-borne, aircraft instruments and satellites, where applicable, are presented in companion papers along with analysis of VOC composition at Beltsville (Doughty et al. 2012) and Edgewood (Halliday et al. 2013). This study addresses the following questions:

1. How do satellite-derived ozone column estimates compare to the Beltsville and Edgewood soundings? OMI total ozone and the OMI/MLS trajectory-enhanced tropospheric ozone residual (TTOR) products are evaluated.
2. What are the relative influences of processes like stratospheric intrusion and pollution transport on temporal ozone variability and vertical structure? This question is addressed with Laminar Identification (Thompson et al. 2007a), Air Quality Indices and tracers from radiosondes (relative humidity) and aircraft, CO and NO_y_.
3. In the case of Beltsville, how do July 2011 ozone structure and meteorological influences compare to 2006–2010, for which summertime sounding data are available? Statistics from LID and standard climatological indicators are analyzed.

The second section describes data sources and methods of analysis. The third section presents results and discussion related to Questions 1 and 2 above; the fourth section covers analyses related to Question 3. The fifth section is a summary.

## Measurements and methods of analysis

### Ozone observations and data processing

Ozone soundings in support of DISCOVER-AQ were made at Beltsville and Edgewood. All sounding data are public at http://www-air.larc.nasa.gov/cgi-bin/arcstat-d. The Beltsville soundings were made with a combination of Droplet Measurement Technologies (DMT) electrochemical concentration cell (ECC) ozonesondes and RS-92 radiosondes in 2011; the DMT sonde that was adopted at this site in 2010 is essentially the same instrument as previously supplied by the ENSCI Corporation. Ozonesondes at the Howard University Research Center (HURC) Beltsville site have been launched since 2004, as described in Morris et al. (2007), Thompson et al. (2007a), Thompson et al. (2007b) and Yorks et al. (2009). The soundings use a 0.5 % buffered KI solution, that when combined with the ENSCI instrument, is shown in laboratory and field tests (Smit et al. 2007; Deshler et al. 2008; Smit (ASOPOS) 2011) to be accurate throughout the troposphere at ~5 %.

The Edgewood ozone and pressure-temperature-humidity (PTU) soundings were made using DMT ozonesondes (Thompson et al. 2000; Johnson et al. 2002; Thompson et al. 2007c) coupled to Intermet IMet-1 radiosondes. A 1 % buffered KI solution was employed which tends to produce ozone readings that are 5–10 % too high. The sonde readings were corrected, based on the performance of ENSCI sondes in a test chamber (Smit et al. 2007; cf Fig. 1 in Thompson et al. 2011; Smit (ASOPOS) 2011); the correction appears in Martins et al. (2013).

**Fig. 1:**
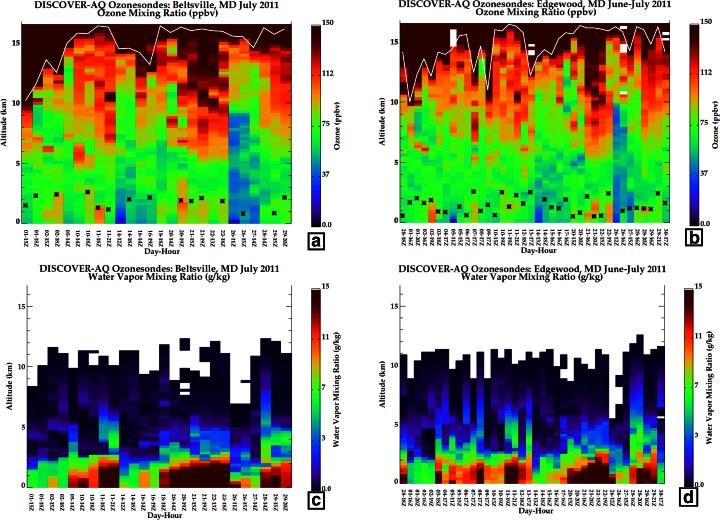
Curtains of ozone mixing ratio (scale in ppbv) for segments averaged over 0.25 km from soundings taken during July 2011 DISCOVER-AQ over **a** Beltsville, MD; **b** Edgewood, MD. The tropopause based on an ozonopause (see text) appears in *white*. The boundary layer height (BLH) is given by *Asterisk*. Curtains of water vapor mixing ratio (scale in g/kg) for segments averaged over 0.25 km from soundings taken during July 2011 DISCOVER-AQ over **c** Beltsville, MD; **d** Edgewood, MD

Total ozone column is obtained by integration of the sounding to burst, ~10–15 hPa in most cases, with a climatological add-on based on OMI, MLS and sonde profiles (McPeters and Labow 2012). Two other ozone column amounts are reported here: ozone within the boundary layer (BL); ozone integrated to the tropopause, designated TTOC, total tropospheric ozone column. The top of the BL is defined according to the altitude at which the thermal lapse rate first exceeds 7 K/km (Martins et al. 2013; see also Heffter 1980). The column amounts are given in Dobson Units (DU); 1 DU = 2.69 × 10^16^ molec/cm^2^.

For the tropopause, an ozonopause criterion is employed, similar to that used by Browell and coworkers (Browell et al. 1996; Fenn et al. 1999). Following the ozone mixing ratio from troposphere toward the lower stratosphere, the largest gradient over a 0.5 km interval is taken as the tropopause as long as ozone above it remains at 100 ppbv or more. The ozonopause (Fig. 1a, b) is typically 1–2 km lower than a thermal-lapse-rate tropopause (not shown) during DISCOVER-AQ in 2011. Exceptions to a well-defined ozonopause occur when sharply-defined layers of high-ozone air appear within the troposphere below the stratosphere-down ozonopause, ie in 3–4 DISCOVER-AQ soundings a “double tropopause” appears (cf Thompson et al. 2007a, b; Yorks et al. 2009).

Surface ozone data at the HURC Beltsville site is obtained from a TEI 49i ozone analyzer that is operated by MDE. At Edgewood, surface ozone readings were taken from the Penn State University Nittany Atmospheric Trailer and Integrated Validation Experiment (NATIVE), with a TEI 49c analyzer. We also use ozone profiles from the NCAR 4-channel analyzer, along with NO_y_ from the same instrument on the P-3 (Table 1); these were measured below 5 km over Edgewood and on spirals below 2 km over Beltsville. The P-3 CO profiles are taken from the NASA Langley Differential Absorption Carbon Monoxide (DACOM) instrument (Table 1).

### Satellite data, data analysis and ancillary measurements

OMI total ozone column from the TOMS v8.5 method is used for total ozone comparisons. For a tropospheric ozone column estimate, we use the TTOR (Schoeberl et al. 2007; Doughty et al. 2011) because it is well-characterized in the mid-Atlantic region (Normile et al. 2014, in review). TTOR data are available for about half the days of July 2011 at the website: ftp://hyperion.gsfc.nasa.gov/pub/aura/tropo3. The TTOR product is assumed to represent column ozone from the surface to 200 hPa. Thus, comparisons with integrated ozone in the soundings use a smaller column than the TTOC that typically extends above 13 km (~150 hPa).

Laminar identification analysis of segments within the free troposphere (FT) for each sounding (LID, Thompson et al. 2007a, b; Luzik 2009; Yorks et al. 2009; Thompson et al. 2010; Thompson et al. 2011) have been used to determine a budget of likely origins: BL, stratospheric ozone, vertically mixed regional pollution. The LID technique, based on a formalism introduced by Teitelbaum et al. (1994), uses normalized profiles of ozone and potential temperature anomalies to infer the presence of a wave and its associated mechanism. A lamina that is affected by a displacement designated as a Gravity Wave (GW) represents vertical mixing. For summer conditions in the mid-Atlantic, this is likely to occur during deep convection or mixing between the BL and lower free troposphere (LFT). Horizontal displacements, associated with a Rossby Wave (RW) designation, usually represent advection of stratospheric air or ozone pollution transported from upwind. Stratospheric influences in northeastern North American soundings during INTEX-A (Singh et al. 2006) are described in Thompson et al. (2007a, b) where ~25 % of summertime tropospheric ozone from soundings originated from the stratosphere. Detailed observations of stratospheric influence in summertime Beltsville soundings (2004–2007) appear in Yorks et al. (2009). Ozone in vertical layers that do not correspond to BL, GW or RW are labeled as “Residual,” corresponding to air parcels containing background air which is aged enough that dynamical origins cannot be distinguished.

In Section 4, where various meteorological parameters for July 2011 are compared to June-July-August (JJA) of years 2006–2010, anomalies for each year are determined graphically at http://www.esrl.noaa.gov/psd/data/composites/hour/. NCEP/NCAR Reanalysis fields are the basis for the imagery presented here.

## Results for DISCOVER-AQ (July 2011)

In this section satellite-sounding comparisons are presented (Section 3.1) along with interpretations of ozone variability using the meteorologically oriented LID (Section 3.2). The general findings from LID are that convective and advective influences occur at different levels within a single sounding, creating a complex structure. Confirmation of meteorological signatures in the structure of individual profiles appears in Section 3.3 where case studies using aircraft data with the sondes are described in detail.

### Ozone profile overview and satellite product comparisons

#### Sonde profiles and OMI-based satellite comparisons

Day to day ozone vertical structure recorded by the sondes launched in Beltsville (hereafter BV) and Edgewood (hereafter EW) appear in Fig. 1, where mixing ratio, averaged in 0.25 km segments, is depicted. There is only one profile at BV (Fig. 1a) between 3 and 10 July, compared to six at EW (Fig. 1b). Water vapor mixing ratios are also displayed (Fig 1c, d). The period 23–25 July was relatively unpolluted; no aircraft flights or sonde launches were conducted. On most other days sampling at both stations followed the same protocol, with two launches made as the P-3 spiraled down overhead. The repetitive route of the P-3 (http://www.nasa.gov/mission_pages/discover-aq/overview/index.html; see also Fig. 1 in Martins et al. 2013) visited BV first, followed by spiraling over 3 additional sites to the north near Interstate-95 before starting the EW spiral that sampled down to 300 m. The BV and EW sites are ~70 km apart and were normally sampled with a 1.25-h interval.

In general the vertical structure at BV and EW appear similar. At both sites the tropopause layer (denoted by red-brown gradients in Fig. 1a, b), is usually located between 9 and 13 km. Exceptions occurred on 10, 11 and 26 July, when the transition appears to be above 14 km. Inspection of the boundary layer height (BLH; stars in Fig. 1a, b) reveals that EW is usually lower in BLH and higher in ozone (see also Table 2). Low water vapor (Fig. 1c, d) <3 g/kg (relative humidity, RH, <15 %) is a persistent feature, appearing below 5 km in 12 of 14 days sampled at BV and 16 of 30 days at EW.

**Table 2:**
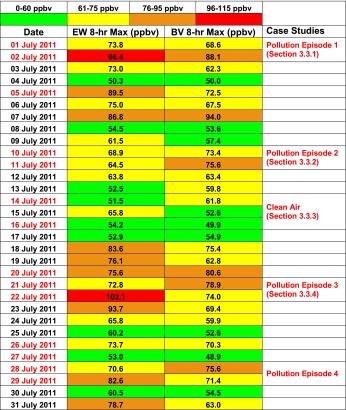
Summary of July 2011 ozone, with P-3 flight days in red (first column), Air Quality code (colors defined in first row)

**Fig. 2:**
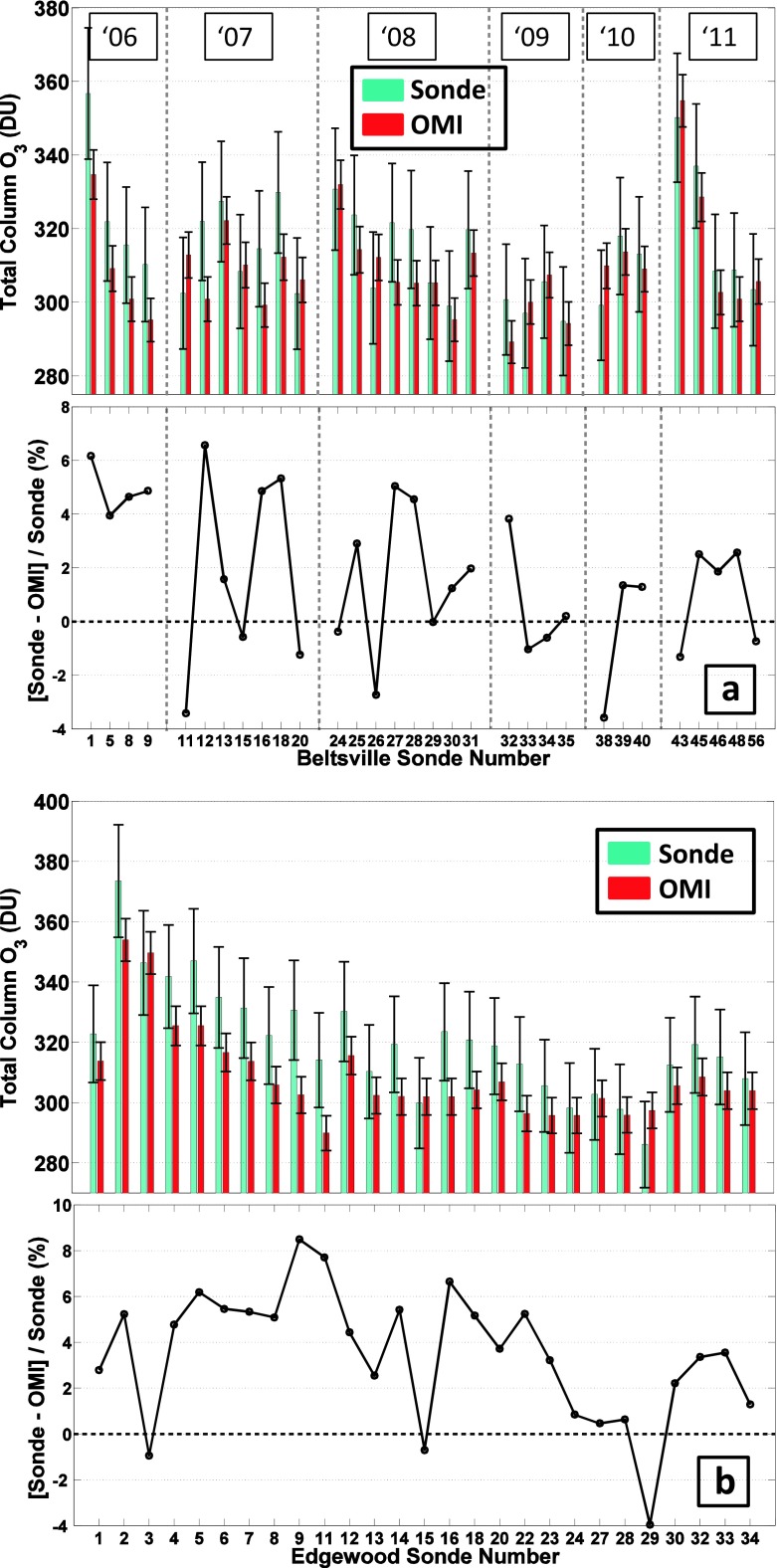
OMI Overpass Comparison. **a** Total column ozone integrated from sonde (*light blue*), as described in text, for launches conducted from 2006 to 2011 over Beltsville, MD, and corresponding OMI overpass (*red*), both expressed in Dobson units (DU). **b** same as (**a**) for July 2011 only, for Edgewood, MD, sondes. Overpass comparison criteria were with 150 km and 3 h of launch. *Error bars* are 1-sigma. In *lower panels* no errors are given because the OMI reference is assumed absolutely correct

In Fig. 2 total integrated ozone from the sondes and the OMI overpass column amounts are compared. In Fig. 2a 6 years of summer data are displayed with a coded launch number (Table 3) gives the corresponding launch date. The requirements of the sonde reaching 20 hPa and a 150-km OMI overpass distance from launch site lead to only five BV coincidences (lower right in Fig. 2a) and 27 for EW (Fig. 2b) in July 2011. The error bar of OMI is 2 %, specified in black bars in the upper frame of each figure. The uncertainty in the sonde integral (aqua) is 5 % (recommended in Smit et al. (2007) and Smit (ASOPOS) (2011)). The lower frames in Fig. 2a and b are % differences between sonde and OMI from [total O_3_(sonde)-total O_3_(OMI)]/total O_3_(sonde).

**Table 3:** Key to profile labelsfor sondes at Beltsville (BV) and Edgewood (EW)

BV Sonde	Year	Month	Day	Time (UTC)
1	2006	6	30	1827
2	2006	7	16	1828
3	2006	7	18	1753
4	2006	7	20	1849
5	2006	8	2	1918
6	2006	8	15	1756
7	2006	8	19	1806
8	2006	8	25	1832
9	2006	8	28	1817
10	2007	6	27	1812
11	2007	7	9	1813
12	2007	7	10	2023
13	2007	7	14	1806
14	2007	7	16	1746
15	2007	7	17	1844
16	2007	7	19	1816
17	2007	7	25	1814
18	2007	7	26	1823
19	2007	7	28	1812
20	2007	8	2	1832
21	2007	8	4	1821
22	2007	8	5	1725
23	2007	8	6	1828
24	2008	7	2	1827
25	2008	7	8	1924
26	2008	7	12	1809
27	2008	7	17	1838
28	2008	7	18	1840
29	2008	8	4	1841
30	2008	8	5	1822
31	2008	8	18	1817
32	2009	6	25	1841
33	2009	7	15	1844
34	2009	8	4	1827
35	2009	8	10	1758
36	2009	8	18	1837
37	2010	6	18	1811
38	2010	6	20	2020
39	2010	6	21	2014
40	2010	8	9	1746
41	2010	8	10	1754
42	2010	8	21	1801
43	2011	7	1	1501
44	2011	7	1	1853
45	2011	7	2	1850
46	2011	7	10	1831
47	2011	7	11	1800
48	2011	7	11	2134
49	2011	7	14	1800
50	2011	7	16	1921
51	2011	7	20	1915
52	2011	7	21	1522
53	2011	7	21	1941
54	2011	7	22	1819
55	2011	7	26	1543
56	2011	7	29	1524
57	2011	7	29	2009
EW Sonde	Year	Month	Day	Time (UTC)
1	2011	6	28	1807
2	2011	7	1	1612
3	2011	7	1	2010
4	2011	7	2	1602
5	2011	7	2	1944
6	2011	7	3	1824
7	2011	7	4	1717
8	2011	7	5	1516
9	2011	7	6	1716
10	2011	7	7	1758
11	2011	7	8	1706
12	2011	7	9	1751
13	2011	7	10	1542
14	2011	7	10	1933
15	2011	7	11	1923
16	2011	7	11	2132
17	2011	7	12	1814
18	2011	7	13	1706
19	2011	7	14	1819
20	2011	7	15	1655
21	2011	7	16	1607
22	2011	7	17	1648
23	2011	7	20	1535
24	2011	7	20	2032
25	2011	7	21	1631
26	2011	7	21	2050
27	2011	7	22	1507
28	2011	7	22	1938
29	2011	7	26	1654
30	2011	7	27	1536
31	2011	7	28	1605
32	2011	7	28	2019
33	2011	7	29	1638
34	2011	7	29	2121
35	2011	7	30	1756

**Fig. 3:**
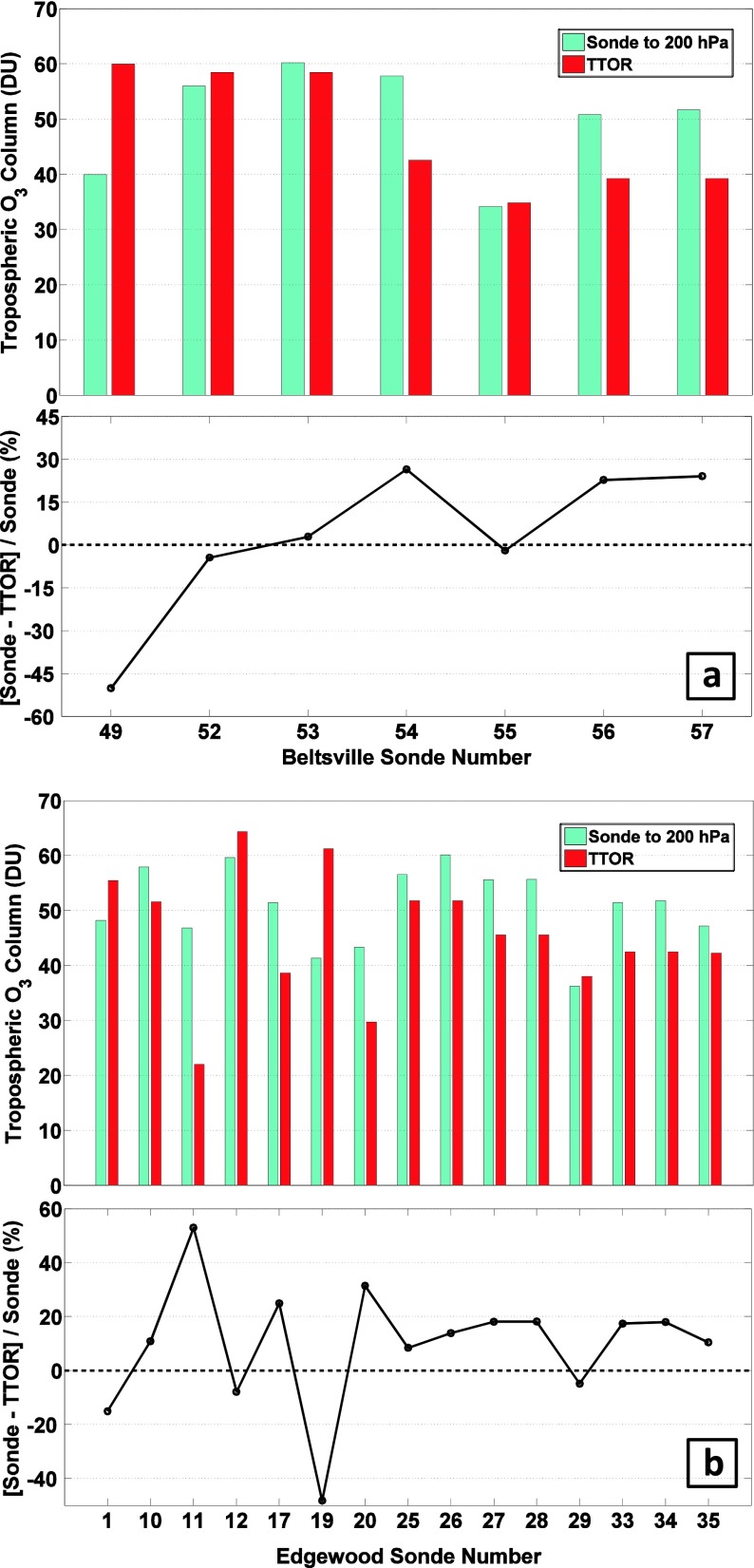
TTOR Comparison. **a** Tropospheric (surface to 200 hPa) ozone columns (*light blue*) in Dobson units [DU] for Beltsville, Maryland, derived from ozonesonde launches in 2006 to 2011. TTOR (trajectory-enhanced tropospheric ozone residual) product based on an OMI-MLS difference (*red*), is compared. **b** same for Edgewood, Maryland, during the July 2011 DISCOVER-AQ campaign

In several prior studies (Doughty et al. 2011; Thompson et al. 2012) we compared the TTOR (Schoeberl et al. 2007) tropospheric ozone product based on subtracting a trajectory-mapped MLS stratospheric ozone column from OMI total ozone. To avoid complexities in defining the tropopause, Schoeberl et al. (2007) report TTOR as an integrated ozone column from surface to 200 hPa. The sonde ozone amounts are compared to archived TTOR in Fig. 3. At BV, except for one unusually clean day (14 July, Profile 49) the discrepancy between sondes integrated to 200 hPa and TTOR averages 10 %. As with total ozone column comparisons at EW (24 of 27 profiles in Fig. 2b), the satellite column, TTOR, over EW usually runs low relative to the sondes (Fig. 3b). The mean absolute error is 10 %, similar to BV; however, for 9 of 15 EW profiles TTOR differs from sonde ozone by more than 15 %. The sonde-integrated column at EW for 5 of 15 comparisons exceeds TTOR by 10 DU or more. Given a 5 % uncertainty in the ozone column amounts from the sondes, these discrepancies are statistically significant.

#### Vertical ozone structure–Beltsville vs. Edgewood comparisons

**Fig. 4:**
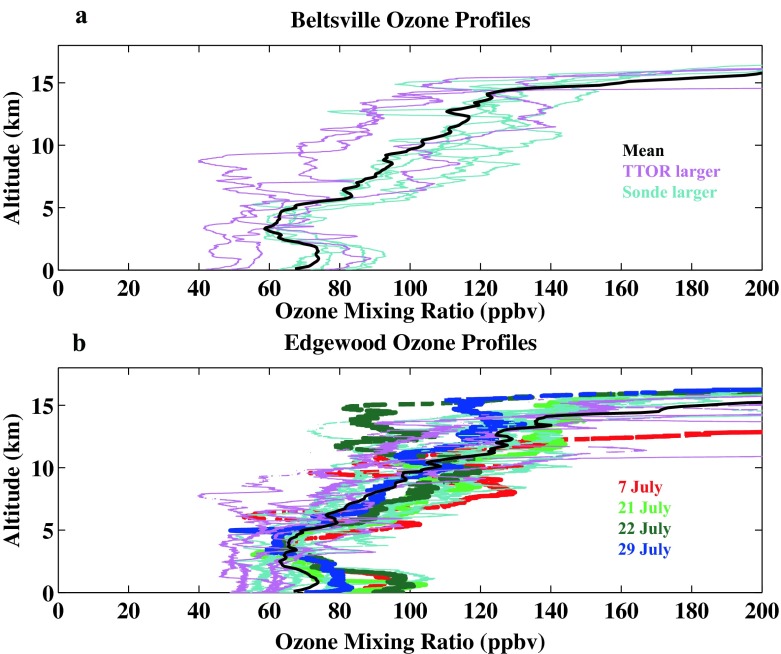
Vertical ozone distribution from soundings over Beltsville (**a**) during DISCOVER-AQ; Edgewood (**b**). The *bold profile* represents the mean over all July 2011 soundings. Individual profiles for the days for which integrated sonde column ozone (to 200 hPa) exceeds the satellite TTOR measurement are in *blue*. For days with TTOR greater than the sonde, profiles are in *violet*. At Edgewood, four days for which sondes exceed TTOR are discussed in text: *red*: 7 July; *light green*, 21 July; *dark green*, 22 July; *blue*, 29 July

Insight into possible causes for sonde-TTOR discrepancy comes from examining individual profiles for days when TTOR is greater than the integrated sonde (Fig. 4, violet lines) and when TTOR is less than the corresponding sounding (blue in Fig. 4a; multi-color in Fig. 4b). The BV deviations from the mean tend to be more uniformly above or below the mean BV profile. Indeed, Martins et al. (2013) (cf Chatfield and Esswein 2012), examining BV and EW profiles from DISCOVER-AQ, found a robust relationship between ozone at 7–10 km and ozone from the surface to 3 km. Correlation coefficients for mean mixing ratios in the two segments was r^2^ = 0.42 (±0.09) for BV, 0.43 (±0.09) at EW. Martins et al. (2013) also point out that for 3–6 km at EW, ozone is not correlated with the surface-3 km and 7–10 km segments. One explanation is that the 3–6 km layer over EW can be decoupled from the surface by processes like the bay breeze (as in dark green profile, Fig. 4b, Stauffer et al. 2012).

**Fig. 5:**
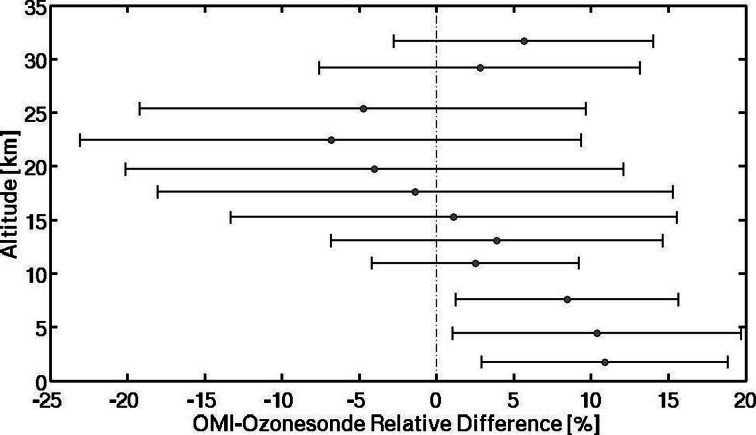
A comparison of ozone profiles from the OMI satellite overpasses with 24 corresponding Edgewood soundings. The coincidence criteria were a median distance and time from the satellite of 300 km and 3 h, respectively. OMI averaging kernel matrices are used to account for changes in OMI vertical sensitivity. The analyses are based on OMO3PR (version 003) data available at http://disc.sci.gsfc.nasa.gov/, with standard quality flags including cloud screening

For EW, the soundings in Fig. 4b display a more complex relationship with the mean profile than for BV. Four soundings in which lower tropospheric ozone exceeded 70 ppbv below 2 km (Fig. 4b), took place on 7, 21 (earlier of two launches), 22 (later launch) and 29 July (later launch). Above 6 km, these soundings also display >70 ppbv mixing ratio but not consistently. The 22 July (dark green) and 29 July (blue) profiles are mostly less than the mean above 6 km. Conversely, two profiles (light blue in Fig. 4b) for which the sonde column exceeds TTOR are less than the mean near the surface. Thus, it appears that the known difficulty in detecting BL ozone is not necessarily the reason for large TTOR-sonde discrepancies (Fig. 3; Reed et al. 2013). The relationship between EW sondes and ozone “profiles” deconvolved from corresponding OMI overpasses presents something of a paradox (Fig. 5). First, for the sonde-OMI coincidences within a 3 h, 300-km window, the tropospheric segment of OMI exceeds the sonde whereas in the mid-stratosphere the opposite holds. Second, no statistically vertical gradient in OMI-sonde appears in the troposphere.

### Meteorological indicators and ozone variability

**Fig. 6:**
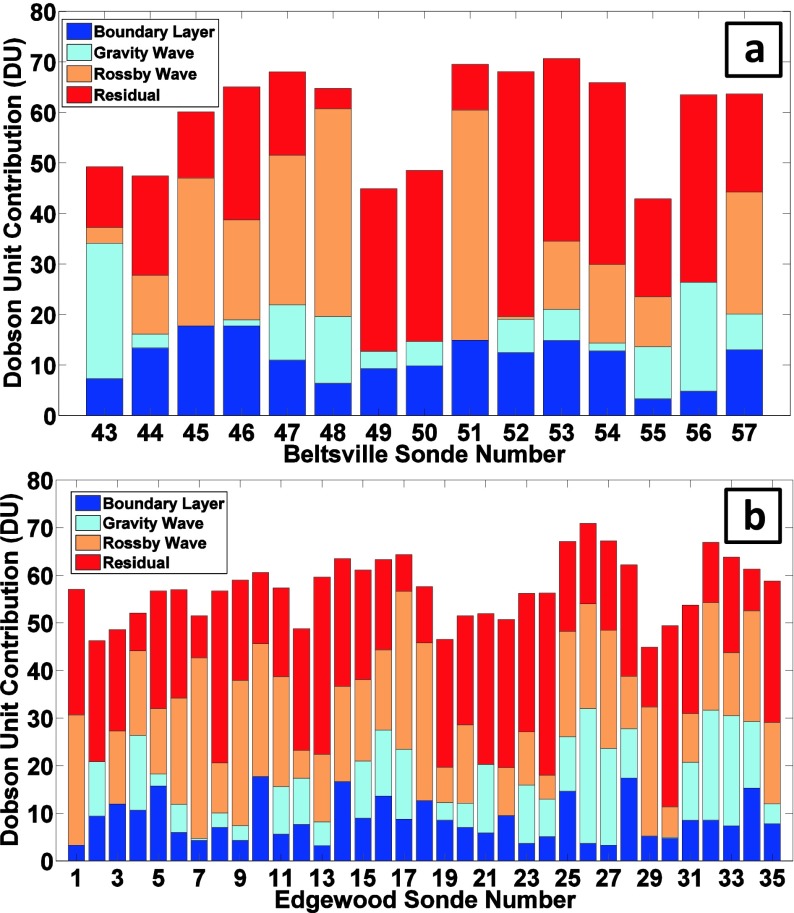
LID Ozone Budgets for DISCOVER-AQ ozonesondes. **a** Tropospheric ozone budgets for Beltsville, Maryland, based on the Laminar Identification (LID) method, as described in (Thompson et al. 2007a, b; 2010) during DISCOVER-AQ, July 2011. Each *bar* represents total tropospheric ozone column (TTOC, to the tropopause depicted in Fig. 1) in Dobson units (DU). **b** as for (**a**) over Edgewood, MD, in July 2011. The *dark blue* column corresponds to the amount of ozone within the boundary layer (BL). The total amount of ozone in segments identified as influenced by gravity waves (GW) by LID is *light blue*. The total amount of ozone in segments identified by LID as influenced by Rossby waves (RW) is given in *orange*. The residual amount of column ozone, in layers that are not in the BL or affected by GW or RW, is *red*. The latter includes ozone that is recently (up to ~ 7 days, as described in Teitelbaum et al. (1994) and Thompson et al. (2007a; 2010)) advected into the region or is considered a background amount with origins too old to be determined by LID. The number of soundings with LID analysis exceeds those in Figs. 2 and 3 where only soundings near Aura overpasses are illustrated

In Fig. 6 the LID analyses for DISCOVER-AQ show considerable day-to-day variability at both BV (profiles 52–54 in Fig. 6a) and EW (profiles 25–28 in Fig. 6a), even within a few hours at each site, e.g. on 21 and 22 July. Edgewood soundings, with fewer gaps over July and generally similar tropospheric columns to those at BV, give an overview of the ozone situation. As for the profile structure at EW (Fig. 4b), examination of ozone budget segments in Fig. 6b, indicates that discrepancies between TTOR and sonde column amount do not follow a single pattern. For 21 July (earlier launch, profile 25) and 22 and 29 July (later launches, profiles 28 and 34), BL ozone exceeds 15 DU. The other 21, 22 and 29 July EW profiles (26, 27, 34) are low in BL ozone with >20 DU ozone corresponding to GW segments (Fig. 6a). For the earlier 22 and 29 July profiles, launches were made before there was much ozone mixed into the BL although there was quite a bit of ozone above it. Over EW, layering in the latter part of 21 July was distinct from other sites sampled nearby by aircraft. He et al. (2014), using DISCOVER-AQ aircraft data, point out that the expected ground level ozone (~100 ppbv) did not materialize on 21 July episode (Section 3.3.4).

### Case studies on chemical variability

Table 2 is a compilation of surface ozone statistics including Air Quality Index color codes and a list of representative pollution periods during DISCOVER-AQ. July 2011 started with a relatively low tropospheric ozone column (45 and 48 DU for the 1 July EW soundings in Fig. 6 profiles 2 and 3, 47 and 49 DU for the corresponding BV columns, profiles 43 and 44 in Fig. 6), followed by a pollution episode that developed on 2 July. Table 2 shows that on 2 July surface ozone exceeded 90 ppbv at both sites, but Code Red, defined by a 95 ppbv limit over 8 h, appeared only at EW (Table 2). No Code Red violations occurred at BV in July 2011. A second polluted period prevailed on 10–11 July (BV profiles 46–48, EW profiles 31–34), followed by 9 days of more moderate conditions. Although it was not characterized by the highest surface ozone (78–90 ppbv ozone when the sondes were launched), a third high-ozone period coincided with the highest July tropospheric ozone columns, > 70 DU on 21 July, at BV (profiles 52 and 53) and EW (profiles 25 and 26, Fig. 6). The second EW Code Red occurred on 22 July (Table 2). The fourth elevated ozone period took place on 28 and 29 July (Fig. 6 with profiles 56 and 57 for BV, 31–34 for EW).

In Sections 3.3.1–3.3.4 the first three pollution episodes and one low-ozone “background” period (Table 2) are described with meteorological conditions, LID analyses, and variability in O_3_, H_2_O, CO and NO_y_ profiles (below 4.5 km for the latter two measurements). A summary appears in Table 4. Pairs of ozone and RH profiles for days of sonde launches at both BV and EW are analyzed. In general the aircraft ozone and the sonde measurement are in close agreement. At both BV and EW the BL is often decoupled from the FT; aircraft CO and NO_y_ profiles up to 2 km (BV) and 4.5 km (EW) display broadly similar vertical structure but hour-to-hour differences in stable layers (laminae) and other small scale features often differ.

**Table 4:** Summary of case study aircraft data below 2 km

Dates	Summary of variations within period
Pollution Period #1, 1–2 July	Starts with BV CO/NO_y_ higher at BV than EW. Transitions to similar CO/NO_y_ later on 1 July before 2 July becomes Code Red at EW with NO_y_ ~8 ppbv at EW compared to BV 5 ppbv.
Pollution Period #2, 10–11 July	Ozone similar on 10 July, but BV in BL has lower CO and higher NO_y_ than EW. On 11 July BV ozone 20 % greater in BL than EW. CO/NO_y_ higher at BV, presumably due to shallower BL, less ventilation at BV
Clean Period, 14–16 July	At BV and EW, ozone similar but BV CO higher and NO_y_ about twice as high as EW.
Pollution Period #3, 20–22 July	20 July—BV more polluted with 50 ppbv more CO, then transitions to EW more polluted on 21 July, when ozone ~110 ppbv at 1 km, exceeded BV by 30 ppbv. On 22 July ozone 2× higher at EW than BV with later profile EW CO 10 % higher than BV; BV NO_y_ 25 % higher than EW.

#### Pollution episode 1: 1–2 July

**Fig. 7:**
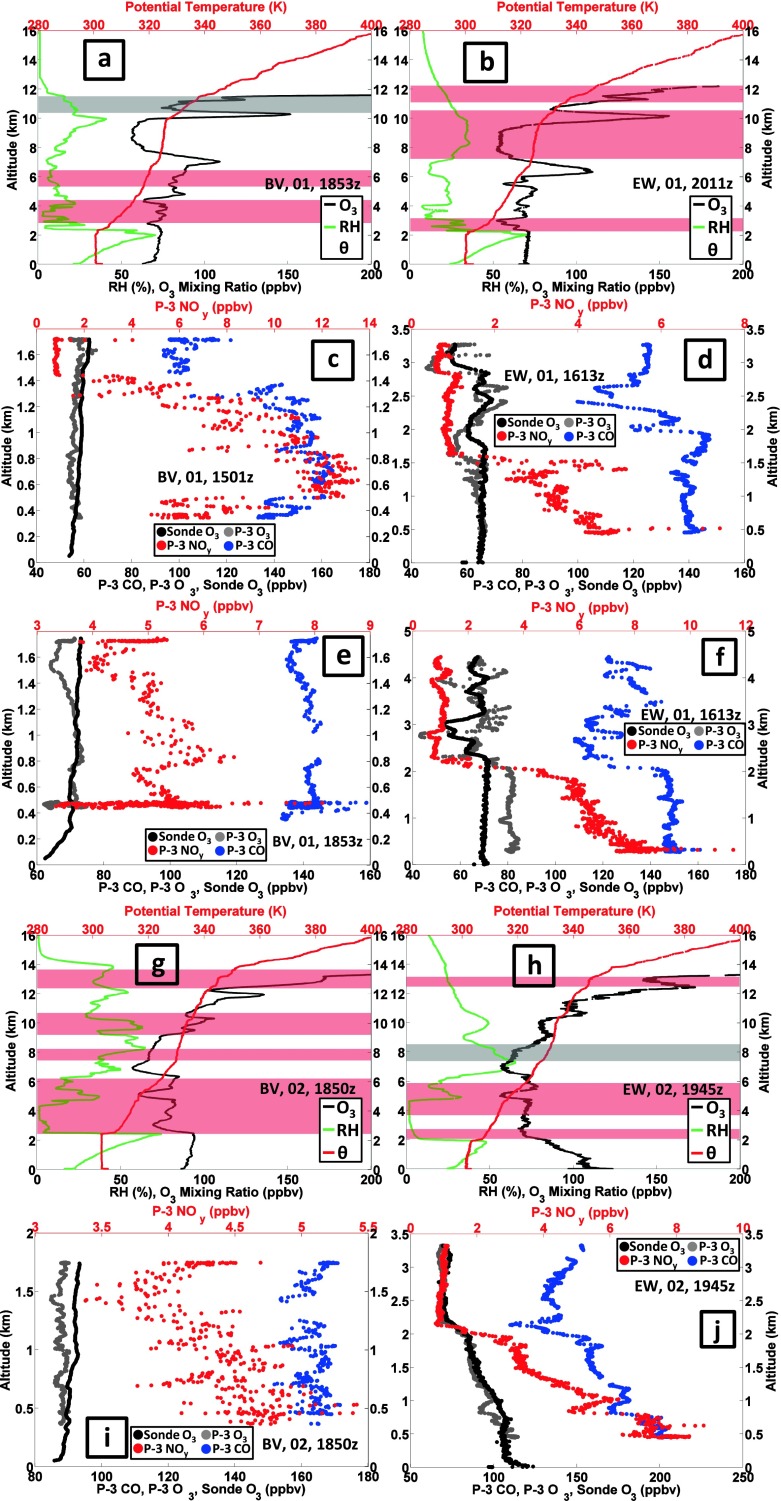
Sounding and aircraft profiles for 1–2 July. Sounding panels display ozone, RH, potential temperature and segments assigned to RW (*light red*) and GW (*gray*). Aircraft ozone, CO, NO_y_ profiles are from P-3 spirals. Note different aircraft altitude ranges (lower at BV)

On 30 June a cold front passed through the DISCOVER-AQ sampling area. With a high pressure system well-established by 2 July, 1 July could be characterized as a transition day (Oyola et al. 2014, in review). On 1 July a relatively low tropopause layer is indicated by the ozonopause (white lines in Fig. 1a, b). The earlier profiles at both BV (Fig. 1a) and EW (Fig. 1b) on 1 July are quite dry above 5 km as displayed in the corresponding H_2_O profiles (Fig. 1c, d). In the early profiles, GW activity is indicated between 5 and 10 km (not shown), suggesting vertical mixing. At BV surface ozone is ~55 ppbv; in ~4 h, the sounding (Figs. 1a and 7a) taken during the 2nd aircraft spiral show that BV near-surface ozone increased to ~70 ppbv. A very dry layer at 3–4 km that is associated with RW in the first sounding remains in the second sounding (lower red shaded band in Fig. 7a). The dryness suggests stratospheric origins, an interpretation that is consistent with tracer data from the P-3. Although aircraft sampling over BV is restricted to less than 2 km (Fig. 7c), the earlier spiral displays a sharp decrease in CO and NO_y_ at 1.3–1.4 km. Above 1.4 km the CO mixing ratio drops from the first aircraft spiral, from a mean 140 ppbv in the BL to ~100 ppbv (Fig. 7c), a very low value for mid-latitude continental FT air (Browell et al. 1996). In the second BV aircraft spiral (Fig. 7e), NO_y_ and CO are well mixed in BL and LFT. In Fig. 7e NO_y_ is 4–5 ppbv and CO is uniformly 135–145 ppbv from ~0.3 km to 1.8 km.

On 1 July at EW, in the second sounding (Fig. 7b), there is a locally dry layer (RH ~10 %) at 6–7 km that coincides with a >100-ppbv ozone layer at the lower-altitude end of an extended RW segment (red shading from ~7 to 10 km Fig. 7b). This EW sounding on 1 July mostly resembles the 2nd BV profile of 1 July (Fig. 7a), with a thin layer at 10 km exceeding 150 ppbv at peak and a ~60 ppbv layer (moister than BV) at 8–10 km. P-3 aircraft spirals over EW begin at 3.2 km or above (Fig. 7d, f). As in the first BV spiral (Fig. 7c), CO over EW in the LFT is lower than in the BL, with a minimum ~110 ppbv at 2–2.5 km (Fig. 7d). This CO feature corresponds to a dry segment in the EW sounding (RH < 10 %, Fig. 7b) and 1 ppbv NO_y_ above 1.5 km in the P-3 spiral over EW (Fig. 7d); stratospheric origins are indicated. Thus, stratospheric influences are present even in the midst of a pollution episode; note the significant RW fraction in EW sonde 3 (later 1 July) in Fig. 6b. The second P-3 spirals record moderately high BL pollution over BV and EW (Fig. 7e, f), e.g. 7–10 ppbv NO_y_ below 0.5 km at EW.

On 2 July, a day designed for overflying a developing pollution episode (Table 2), there was only a BV launch during the second P-3 circuit (Fig. 7g, i). The BV ozone profile (Fig. 7g) displays 4 red-shaded bands (RW) at altitudes with locally high ozone and moister air (RH ~50 %, compared to <30 %) than the day before. Isolated from the bay breeze (Stauffer et al. 2012) on 2 July, pollution does not develop at BV. Instead, the P-3 sampling over BV (Fig. 7i) reveals moderate, well-mixed pollution (CO = 150–170 ppbv, O_3_ = 80 ppbv, 3.5–5.5 ppbv NO_y_) from 0.38 to 1.8 km.

The first EW sounding on 2 July (at 1802 UTC, not shown) was similar to the second set of BV and EW profiles on 1 July. However, there was a GW feature over EW in the sounding from 7 to 11.5 km, indicating vertical mixing, and a RW segment from 2 to 6 km, suggestive of advection. In the later EW profile (GW, gray shading at 8 km in Fig. 7h) the mixing may have contributed to enhanced ozone above 10 km. Horizontal transport identified by Stauffer et al. (2012) as a bay-breeze on 2 July leads to 120 ppbv surface ozone at EW and an 8-h maximum of 96 ppbv (Table 2). The corresponding P-3 tracer spirals over EW (Fig. 7j) show ozone >100 ppbv near the surface with 200 ppbv CO and 4–8 ppbv NO_y_ below 1.5 km. Above 2 km, the P-3 records CO < 120 ppbv and NO_y_ < 1.5 ppbv (Fig. 7j) and the EW sounding (Fig. 7h) shows 70 ppbv ozone and very dry air up to 5 km.

#### Pollution episode 2: 10–11 July

**Fig. 8:**
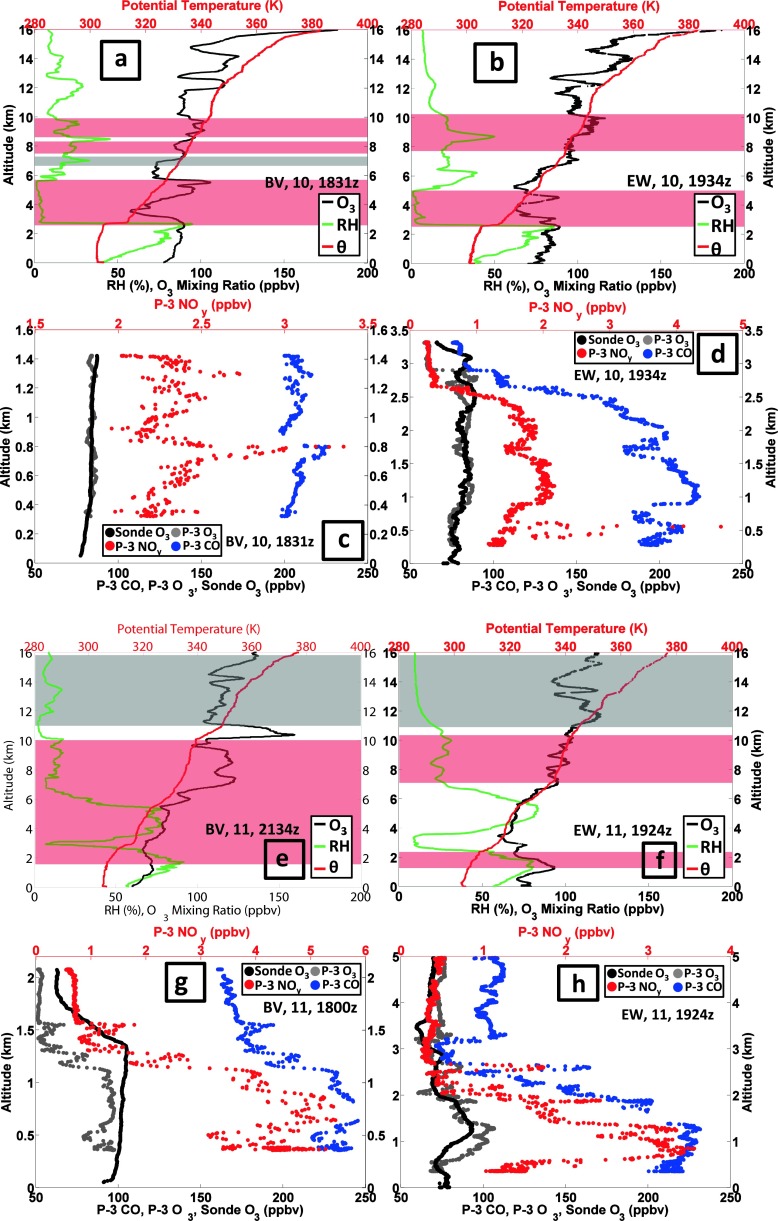
As for Figure 7 with sounding and aircraft data for 10–11 July 2011

A moderate ozone episode took place on 10–11 July, sampled by aircraft and launches at each station (Fig. 8). Only at EW were there two 10 July soundings; they do not diverge much from the 10 July BV profile (Fig. 8a) that took place an hour before the later EW launch (Fig. 8b). The BV ozone aircraft profile (1831 UTC, Fig. 8c) is nearly identical to the corresponding sounding in the BL, but CO is ~200 ppbv below 1.5 km with 2–3 ppbv NO_y_. There is considerable fine structure in ozone and θ, with GW (gray shading) or RW (red shading) zones in all the 10 July soundings. Over EW (Fig. 8b) between 3 and 5 km, moderate ozone in a very dry layer corresponds to a RW (red) signature, indicative of advection. The dryness suggests stratospheric origins, as does the P-3 profile (Fig. 8d) with 80 ppbv CO, which is approximately half the normal LFT CO concentration for the mid-Atlantic.

There were four soundings on 11 July, one BV and one EW sounding each in the 1800–1930 UTC period and again after 2100 UTC. As in the 1–2 July profiles there is a very dry layer at 3 km in all of the 11 July profiles. The earlier BV sounding has ~100 ppbv at the surface (not shown) whereas the corresponding EW profile is 80 ppbv (Fig. 8f), overlaid by a layer of moderate ozone, with a higher ozone layer above the BL at 1–2 km. The BV P-3 profile (Fig. 8g) is polluted, with 90–100 ppbv ozone, CO > 225 ppbv and NO_y_ > 5 ppbv down to 0.35 km. Both EW aircraft profiles (Fig. 8h is the later one) resemble near-surface BV CO and NO_y_ pollution. In a dry layer at 3 km, CO over EW drops to 80 ppbv with 0.5 ppbv NO_y_, suggesting stratospheric influence (RW band at 2 km in the sounding, Fig. 8f). As during the 1–2 July pollution episode, stratospheric influences persist within pollution.

#### Cleaner air: 14–16 July

**Fig. 9:**
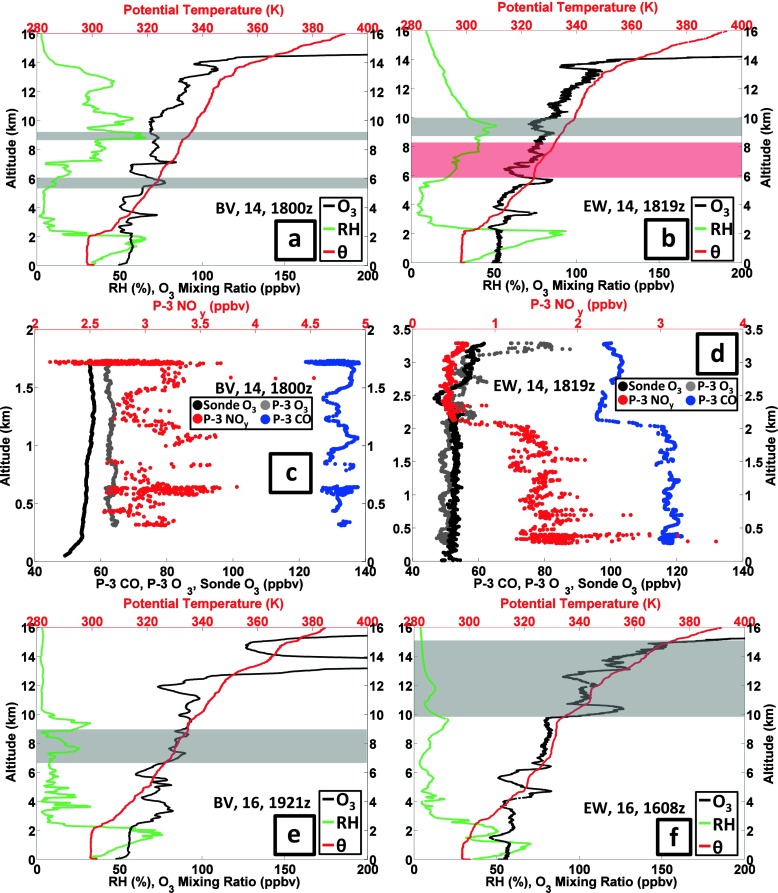
As for Figure 7 with sounding and aircraft profiles for 14 July 2011

The lowest tropospheric ozone columns, 45 DU, were sampled on 14 July (BV profile 49, EW profile 19 in Fig. 6), with the corresponding ozone, θ and RH profiles depicted in Fig. 9. The BV and EW profiles are mostly 50–60 ppbv from the surface to 8 km (Fig. 9a, b). On 14 July the BL is ~2.5 km at both sites, above which is a very dry layer up to ~6 km. An interpretation of stratospheric impact is supported by the RW signature in the EW sounding (Fig. 9b). In Fig. 6b the ozone RW budget for the 14 July EW sounding represents 25 % of the ozone; the prior day, 13 July, more than half the ozone over EW was RW-classified. The air parcels sampled in the 14 July soundings can be considered as a background composition. Besides 50–60 ppbv aircraft ozone the corresponding P-3 spirals (1800–1830 UTC in Fig. 9c, d) display low pollution conditions with 130 ppbv CO below 1.8 km at BV (Fig. 9c). Over EW (Fig. 9d) CO is 120 ppbv from surface to 2 km, dropping to 100 ppbv from 2 to 3.4 km. The 16 July soundings (Fig. 9e, f) are similar to those for 14 July but they display lower RH in much of the troposphere (Fig. 1c, d).

#### Pollution episode 3: 20–22 July

**Fig. 10:**
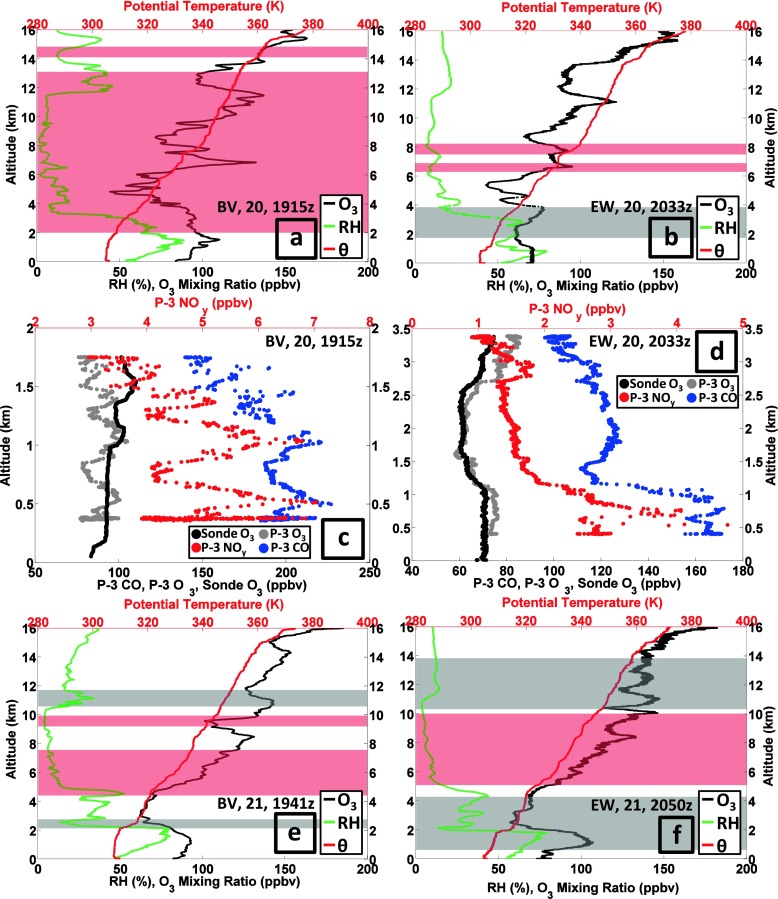

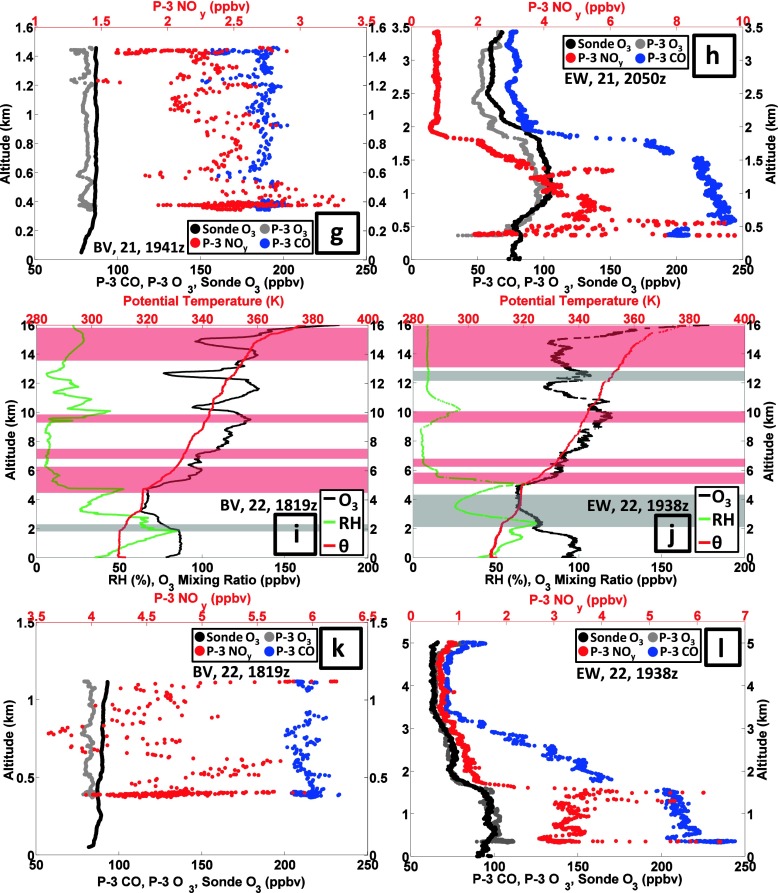
As for Figure 7 with sounding and aircraft profiles for 20–22 July 2011

Stauffer et al. (2012) classify Edgewood as following an ‘interrupted’ bay breeze; on 19 July the 1-h maximum ozone was 108 ppbv. On 20 July the sole BV sounding (Fig. 10a) was richly layered, with ozone exceeding 100 ppbv at 1.5–2 km, 6–8 km, 10–12 km and 13–16 km; the EW sounding was broadly similar above 5 km (Fig. 10b). Over BV (Fig. 10a), RH was greater above 12 km than from 6 to 11 km, where the RH was <10 %. The moister upper troposphere suggests convective mixing whereas the drier region may have stratospheric origins, consistent with the RW character of the BV ozone profile (red shading in Fig. 10a). Indeed, below 1 km CO displays polluted values, 190–220 ppbv at BV (Fig. 10c) and 170 ppbv at EW (Fig. 10d), with 3–7 ppbv NO_y_. Above 1 km the EW ozone declines to 60 ppbv, increasing again above 2.5 km. At 1.5 km there is a local 110 ppbv CO minimum (Fig. 10d). Above 1.5 km, CO increases to 125 ppbv, then declines to 100 ppbv at 3.5 km. The near-surface concentration of NO_y_ is ~4 ppbv, but declines above 1 km.

Flights on 21 July were made by the P-3, UC-12 and UMD Cessna (He et al. 2014). There were two soundings each at BV and EW; the later profiles (1940–2050 UTC) are depicted in Fig. 10e, f. The 5th Code Orange day, with 8-h maximum 79 ppbv ozone (Table 2), is recorded at BV. Below 1 km during the earlier P-3 spiral over BV (not shown), the highest NO_y_ (14 ppbv) and CO (315 ppbv) concentrations of the DISCOVER-AQ P-3 flights were observed. In the second BV spiral, ozone (CO) is 80 (210) ppbv (Fig. 10g); the NO_y_ concentrations are half the level of 4 h earlier.

In all four 21 July soundings (two depicted in Fig. 10e, f), from 5 to 10 km, ozone increased from the prior day to 100 ppbv with dry conditions. Stratospheric influence is implicated; note a thin dry layer at the top of the BL (Fig. 10e, f). Stratospheric influence over EW is also suggested by CO and NO_y_ drop-offs above 2 km (Fig. 10h). In the EW sounding (Fig. 10f), an extended GW signature (gray shading) implies recent mixing, but the BL is capped at 0.5 km. A 100-plus ppbv ozone layer at 2050 UTC extends above the BL over EW but does not mix down to the surface. The EW 8-h maximum is <75 ppbv so EW remained within the Code Yellow range (Table 2).

On 22 July, there was only one BV launch (Fig. 10i). From the surface readings (Table 2; cf Fig. 10i, j) it is seen that EW and BV surface ozone is reversed from the day prior. BV (Fig. 10k) just missed Code Orange with a 74 ppbv 8-h mean. A situation described by Stauffer et al. (2012) as “interrupted bay breeze” allows EW ozone to build to 90-plus ppbv after a thunderstorm (second launch, Fig. 10j). The 8-h EW surface ozone maximum is 102 ppbv, giving the second July 2011 Code Red day at EW. Above 6 km in the EW sounding (Fig. 10j), a multi-layered high-ozone structure with low RH (Fig. 1d) remains intact for the third day. The P-3 spiral indicates low CO and NO_y_ above 1.5 km over EW (Fig. 10l). Despite the polluted conditions of the late afternoon sampling, the late morning soundings of 22 July (not shown) have a well-defined BL to ~580 m within which ozone is ~60 ppbv, which is relatively clean air. The EW morning sampling on 22 July, with VOC unusually low in anthropogenic species and elevated in biogenics (Halliday et al. 2013), is traced back to rural western Maryland and Pennsylvania. Evidently, a wind shift following the thunderstorm (note vertical mixing signature in GW feature, gray shading in Fig. 10j) allowed the bay-breeze to introduce polluted air before it was interrupted.

## DISCOVER-AQ in the context of Beltsville ozone climatology (2006–2011)

### Satellite comparison overview

**Fig. 11:**
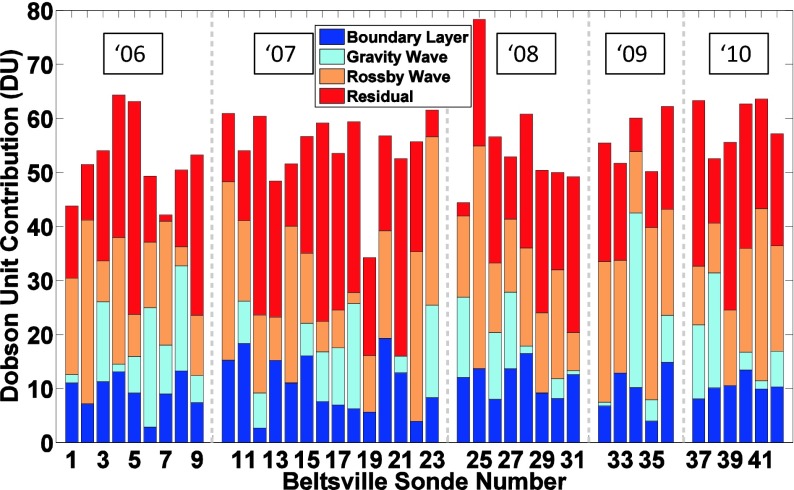
Multi-year LID tropospheric ozone budgets for Beltsville, as in Fig. 6, except that all daytime soundings for JJA 2006–2011 are displayed. Annually averaged BL, GW and RW amounts appear in Table 5

Ozone soundings have been taken at BV for the purpose of studying pollution since 2004 (Thompson et al. 2007a, b). Data from nighttime and early morning launches are not included in satellite comparisons (Fig. 2a) and budget calculations (Fig. 11). Figure 2a shows that for 2006–2011, there are 3–8 overpasses/year with average 3 % offsets. Like the 2011 OMI-sonde differences (Fig. 2), OMI total ozone is usually lower than the BV sondes.

### LID Interannual variability and meteorological indicators

Figure 11 displays TTOC and LID ozone budgets for BV JJA soundings during 2006–2010. A summary of mean TTOC and LID budgets for BV (2006–2011) and EW (July 2011) appears in Table 5. A striking difference between the 2011 TTOC at BV and the prior 5 years is the magnitude of the DISCOVER-AQ TTOC. Although the average TTOC for 2010 and 2011 at BV is essentially the same (59 DU) and <10 % greater than the 2006–2009 mean, the number of TTOC greater than 65 DU in 2011 is unique. Seven of 15 of the BV DISCOVER-AQ sondes (Fig. 6a) have TTOC > 65 DU whereas from 2006 to 2010 only 1 in 41 soundings was that high (Fig. 11). As stated above, 2009 was a low-ozone year at BV. Ozone in the BL (2009 entry, Table 5) is among the lowest in the 6-year BV record, with both GW and RW amounts relatively high. In Thompson et al. (2007a) it was shown that RW segments tend to be higher in years with lower surface pressure, ie with conditions that foster less photochemical ozone formation. We examine this further by looking at meteorological conditions for 2006–2011.

**Table 5:** Summary of LID ozone budgets from Beltsville JJA soundings (2006–2010) and Beltsville (BV) and Edgewood (EW) budgets, July 2011. Ozone is given in Dobson Units (DU)

Year	BL (DU, %)	GW (DU, %)	RW (DU, %)	Residual (DU,%)	Total (DU)
BV, 2006	9.36, 17.8	8.90, 17.0	15.6, 29.8	18.6, 35.4	52.4
BV, 2007	10.7, 19.5	5.70,10.4	15.7, 28.8	22.6, 41.3	54.6
BV, 2008	11.7, 21.2	5.88, 10.6	17.9, 32.3	19.9, 35.9	55.3
BV, 2009	9.74, 17.4	9.12, 13.1	21.9, 39.2	15.1, 27.0	55.9
BV, 2010	10.4, 17.6	7.73, 13.1	17.4, 29.5	23.6, 39.8	59.1
BV, July 2011	11.2, 18.9	7.76, 13.1	16.3, 27.3	24.2, 40.7	59.5
EW, 7/11, All	8.66, 15.2	8.51, 14.9	17.6, 30.8	22.3, 39.1	57.1
EW, 7/11 BV days	9.37, 16.1	11.5, 19.8	15.0, 25.9	22.2, 38.2	58.1

**Fig. 12:**
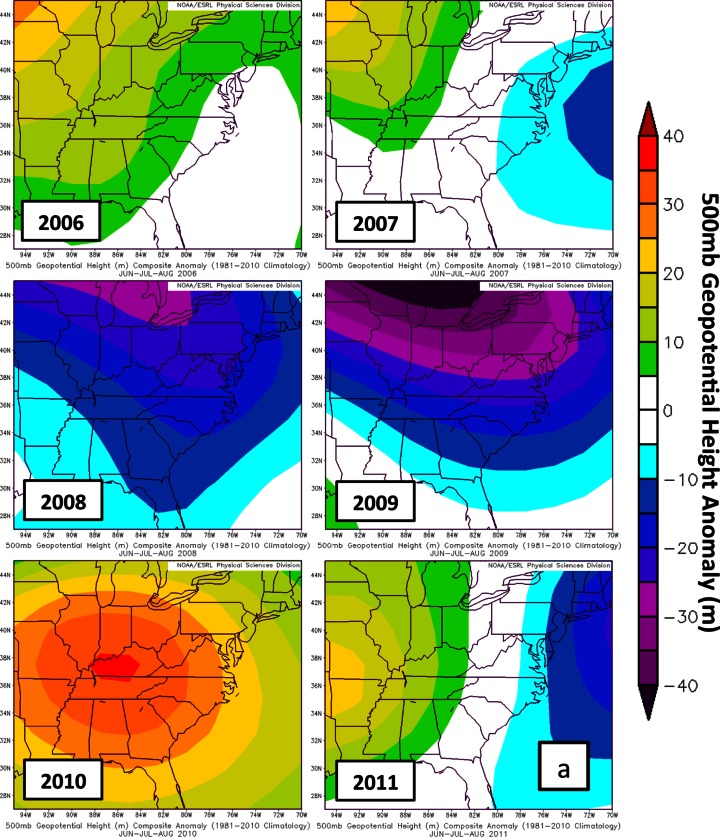

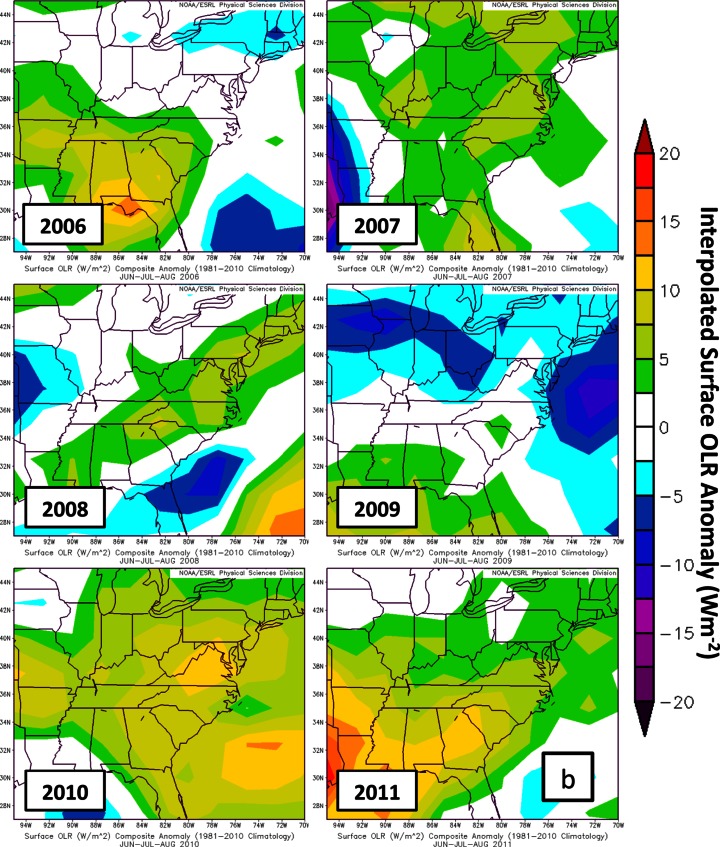

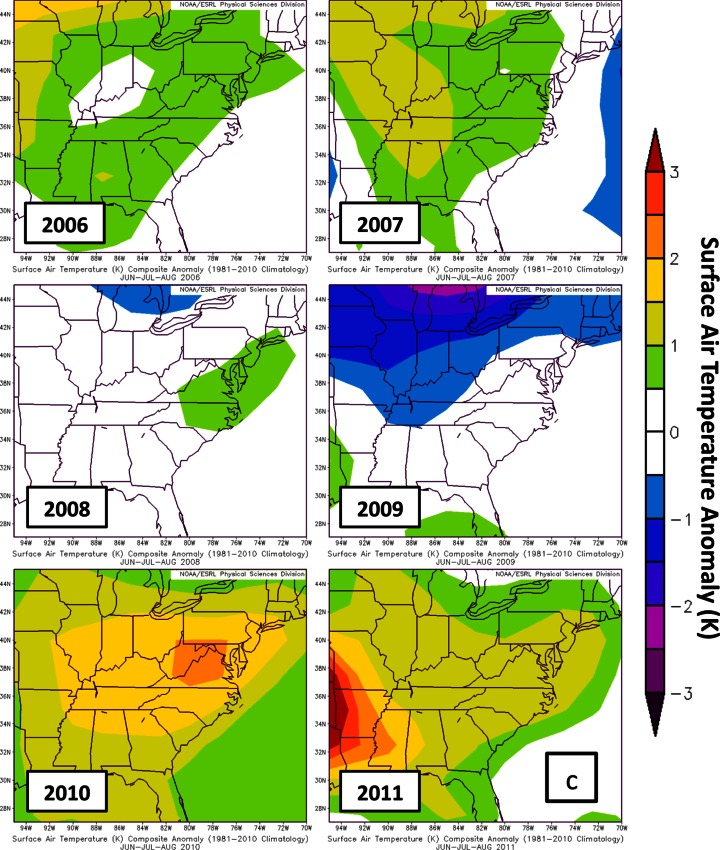
Summary of meteorological variables to describe years 2006–2011 for the months JJA (Refer to Yorks et al. (2009) for statistics for 2004–2007). Data and graphical presentation from the Earth System Research laboratory (ESRL) of National Oceanic and Atmospheric Administration (NOAA). Anomalies relative to 1980 to 2011, for **a** 500 hPa geopotential height; **b** Outgoing Long-wave Radiation (OLR), as a proxy for cloud cover; **c** seasonal surface temperature (warmer or cooler than normal)

Parameters assembled from NCEP/NCAR reanalysis are used to examine large scale variability in the DISCOVER-AQ sampling region (Fig. 12; http://www.esrl.noaa.gov/psd/data/composites/hour/). The 500mb geopotential height anomalies from the 1981–2010 climatological average for JJA are well below normal in 2008 and 2009 (Fig. 12a). During the summer months of 2008 and 2009, a more active wave pattern led to lower 500mb heights (Fig. 12a) and increased stratospheric intrusions, causing enhanced RW signatures at Beltsville in 2008 (17.9 DU average) and 2009 (22.0 DU). The summers in 2006 (9.36 DU ozone, Table 5) and 2009 (9.74 DU) were rainier than normal; enhanced cloudiness, implied from out-going long-wave radiation (OLR) in 2009 (Fig. 12b), resulted in lower BL ozone due to suppressed ozone photochemical production. Warmer than average summers (Fig. 12c) in 2007, 2008, 2010 and 2011 (10.4–11.7 DU at BV) help to explain relatively enhanced BL ozone (Table 5, Fig. 11). Although only July 2011 is available for comparison between EW (8.66 DU in Table 4) and BV (11.3 DU), the July LFT and BL ozone at EW appear to be affected by the bay breeze that leads to lower BL heights (Fig. 1b; see Stauffer et al. (2012)). Thus, EW BL ozone is ~20 % lower than at BV (Table 5) despite the fact that EW *surface* ozone is usually higher (Table 2, Fig. 4). In Table 5 the second EW budget, based on days for which there were BV launches, shows similar RW and Residual ozone at the two sites but a higher GW fraction at EW, implying greater mixing in the free troposphere.

## Summary

Using soundings, aircraft and ground based data from the July 2011 DISCOVER-AQ experiment, we investigated the variability of tropospheric ozone at two suburban locations in Maryland: Beltsville near Washington, DC and Edgewood near Baltimore. In addition, satellite measurements, based on OMI (total ozone) and OMI/MLS (tropospheric column) were compared to the total and partial ozone column measurements from the sondes. Total ozone at both sites (in the case of BV, also including summer soundings from 2006 to 2011) averaged 3 % higher than OMI, not statistically significant. At both locations in July 2011, tropospheric column ozone from the sonde (surface to 200 hPa) was 10 % greater than the corresponding OMI/MLS TTOR product, which is significant. The latter discrepancy is not consistently due to undetected high ozone amounts in the BL (Stauffer et al. 2012; Martins et al. 2013). The DISCOVER-AQ TTOR evaluation is similar to July comparisons for Wallops Island (2005–2010, Normile et al. 2014, in review), although TTOR-sonde offsets are more variable at Wallops.

Laminar analysis of tropospheric ozone column segments that are influenced by convective and advective transport reveal differences in BV and EW. Vertical mixing in the free troposphere was stronger at EW, denoted by the LID Gravity Wave designation (Thompson et al. 2007a), for days when both stations launched ozonesondes and aircraft profiled overhead. Vertical mixing was a persistent signal in EW sondes even though a capped BL over EW implies some decoupling of the BL and LFT (Martins et al. 2013). Approximately half the ozone soundings in the LFT over BV and EW were very dry (RH < 20 %) with low-concentration NO_y_ and CO layers measured by the P-3, suggesting stratospheric influence.

Throughout July 2011, the P-3 ozone, CO and NO_y_ profiles over BV and EW were broadly similar but detailed laminae differed, with higher CO and NO_y_ below 2 km (maximum altitude for P-3 sampling over BV) alternating between the two sites. Differences for CO were usually 20–30 ppbv in the BL but NO_y_ sometimes differed by 50 % (3–4 ppbv). Surface 8-h maxima ozone at the two sites disagreed by 5–25 ppbv on 23 of 31 days, with EW higher on 16 days and BV higher on 7 days. Interannual variability of the tropospheric column LID budgets at BV (2006–2011) is consistent with meteorological patterns. Assuming that RW denotes stratospheric influence, the 25 % of tropospheric ozone column designated as RW over BV and EW in July 2011 appears to be typical for warm, high-pressure conditions in the mid-Atlantic (Thompson et al. 2007b).
